# Regional living conditions and the prevalence, awareness, treatment, control of hypertension at the individual level in Russia

**DOI:** 10.1186/s12889-022-12645-8

**Published:** 2022-01-30

**Authors:** Sergey Alekseevich Maksimov, Yulia A. Balanova, Svetlana A. Shalnova, Galina A. Muromtseva, Anna V. Kapustina, Oksana M. Drapkina

**Affiliations:** National Medical Research Center for Therapy and Preventive Medicine, 10 bld. Petroverigskiy lane, 101990 Moscow, Russian Federation

**Keywords:** Hypertension, Regional characteristics, Place of residence, Russia

## Abstract

**Background:**

The objective of our study was to investigate the associations of characteristics inherent in large Russian Federation (RF) regions with prevalence, awareness, treatment and control of hypertension at the individual level.

**Methods:**

Regional characteristics were obtained from the official website of the Federal State Statistics Service of the RF. We employed principal component analysis to reduce the dimensionality of data, which allowed defining five integral regional indices. Prevalence, awareness, treatment and control of hypertension were assessed from the data of the cross-sectional stage of ESSE-RF study conducted in 2013–2014. The final sample included 19,791 patients from 12 RF regions. Generalized estimating equations were used to identify the associations of regional indices with prevalence, awareness, treatment and control of hypertension at the individual level, taking into consideration nested data structures (study subjects in the regions).

**Results:**

The index characterizing deterioration of social living conditions and societal marginalization exhibited positive associations with the prevalence of hypertension among men (OR = 1.18; 95% CI: 1.05–1.32) and elderly people (OR = 1.16; 95% CI: 1.02–1.32). Moreover, deterioration in the social environment was associated with a reduction in treatment (OR = 0.76; 95% CI: 0.64–0.90) and control of hypertension (OR = 0.79; 95% CI: 0.69–0.90). Hypertension awareness was directly connected with demographic crisis (OR = 1.13; 95% CI: 1.02–1.25) and augmented industrial development (OR = 1.15; 95% CI: 1.01–1.33) in the regions. The association of regional living conditions with the prevalence of hypertension is relatively weak, compared to predictors at the individual level, but this influence is important for awareness, treatment and control of hypertension.

**Conclusion:**

The study contributed to evaluating the associations of the vital characteristics inherent in population of large RF regions with arterial hypertension prevalence, as well as with awareness, treatment and control of this disease. Our results provided original insights from the standpoint of cardiovascular disease epidemiology in the RF, as well as in the context of investigating the impact of living conditions on population health.

## Introduction

Arterial hypertension is the most significant modifiable risk factor for cardiovascular diseases [1]: its proper management could reduce both cardiovascular and overall mortality rates [2, 3]. In their systematic review, P.M. Kearney et al. demonstrated that the prevalence of hypertension in an age group of 20 + years old worldwide ranges from 28.5% in the countries with a high level of economic development to 31.5% in less developed countries [4]. The authors stated a significant increase (from 25.9% to 31.1%) in the global incidence of hypertension over the period of years 2000–2010. In addition, increases in awareness of this disease (from 41.4% to 46.5%), treatment coverage (from 31.8% to 36.9%), and treatment effectiveness (from 33.9% to 37.1%) were also observed during this period of time.

Many published sources described territorial differences in the prevalence of hypertension within particular countries [5–8]. At the same time, it is worth noting that individual-based characteristics may have a significant impact on the prevalence, awareness, treatment and control of hypertension [9, 10]. Besides, differences in the population structure of places of residence due to varying individual-based characteristics could, to a certain extent, account for geographical differences. However, the results of published studies revealed territorial differences in the prevalence of this disease as well – additionally to the considered individual-based features. Even the results of the Monitoring Trends and Determinants in Cardiovascular Disease (MONICA) project of the World Health Organization implied that population factors in geographic regions explain up to 7–8% of all differences in systolic blood pressure [11]. Several publications have demonstrated that territorial features in the place of residence affect the prevalence, awareness, treatment and control of hypertension [12–16]. However, it should be mentioned that nearly all published sources on hypertension describe rather small areas: there are very few studies of associations at the level of countries or large regions. Among the latter, we should mention the population study of large regions in Columbia [17], along with research conducted in the United States, analyzing the impact of socioeconomic characteristics of states on the possibility of hypertension development at an early age, using the case study of Alcoa employees [18].

It should be emphasized that in a majority of studies, territorial features were expressed via 1–2 independent socioeconomic parameters (Gini index, crime rate, average annual income, etc. [17, 19–21]), or economic parameters (poverty index, deprivation index, etc. [22–24]). We found just a single study with an empirical approach and four latent factors out of 20 territorial parameters, including racial/ethnic composition, socioeconomic status, age composition, family structure, owner occupied housing, and housing stability [13]. Only such empirical studies allow evaluating the entire diversity of factors (besides socioeconomic features) that have an impact on the state of health.

Epidemiology of Cardiovascular Diseases and Risk Factors in the Regions of the Russian Federation study of 2012–2103 revealed regional differences in the prevalence, awareness, treatment and control of hypertension [25]. However, we do not know, whether such differences are also influenced by the regional specificities of the living conditions, in addition to the individual-based characteristics in the structure of regional samples. Such multilevel analysis has not yet been carried out. The objective of our study was to investigate the associations of characteristics inherent in large Russian Federation (RF) regions with prevalence, awareness, treatment and control of hypertension at the individual level.

## Methods

### Sampling procedure

Data for the subsequent analysis were borrowed from the cross-sectional stage of Epidemiology of Cardiovascular Diseases and Risk Factors in the Regions of the Russian Federation (ESSE-RF) study conducted in 2013–2014 in 13 RF regions. Detailed description of the sampling procedure and algorithm of ESSE-RF study was presented in an earlier publication [26]. Sampling was performed via using Kish method providing a systematic multistage random selection of study subjects on a territorial basis from medical institutions. The study was carried out in accordance with the Good Clinical Practice standards and the principles of the Declaration of Helsinki. Written informed consent was obtained from all participants prior to their enrollment in the study. Proportion of examined subjects was about 80%, with some variations across the studied regions.

The master sample included 21,923 subjects 25–64 years old. A subgroup from St. Petersburg (1,588 subjects, 7.2%) was removed from the master sample because of its substantial differences in regional characteristics from remaining 12 regions under study. The city of St. Petersburg is classified as a separate constituent entity of the RF, while other 12 regions represent large territories with both urban and rural areas. At the second step of the sampling protocol, participants with incomplete data or with no information on hypertension status were also eliminated from the master sample (535 subjects, 2.6%). Then we excluded the participants who did not answer the question, “Did a physician or another medical professional ever tell you that you had high blood pressure?” (9 subjects, 0.04%). Hence, the final research sample encompassed 19,791 subjects.

The final sample included no data on income of 248 subjects (1.2%), obesity of 211 subjects (1.1%), marital status of 150 subjects (0.8%), educational level of 16 subjects (0.08%), and smoking status of 14 subjects (0.07%). Since there were few missing data, no analysis of the possible missing data bias was conducted. The imputation of missing data was performed using the k-nearest neighbors algorithm for the input values of gender, age, region and place of residence.

### Blood pressure measurement and definitions

Blood pressure was measured twice in a sitting position, on the participant's right arm freely lying on the table at heart height by automatic tonometer (Omron M3 Expert, Japan) using an appropriately sized cuff. Measurement was carried out after a 5-min rest, with an interval of about 2–3 min in a quiet room. Recorded values of systolic and diastolic pressure were carefully checked and cleared of outliers. The comments of an interviewer on any issues that arose in the course of measuring the blood pressure of respondents were taken into account as well. Then, the average of two consecutive blood pressure measurements was calculated. Cases with solely one blood pressure measurement were excluded from the study. Binary parameters of hypertension, awareness, treatment and control of hypertension were analyzed.

We considered that hypertension has occurred when the following conditions were met: (a) average systolic blood pressure of 140 mmHg or higher; (b) and/or average diastolic blood pressure of 90 mmHg or higher; (c) and/or reporting antihypertensive agents’ intake within the past two weeks.

Subjects were presumed to be aware of their hypertension if they (a) were identified as hypertensive patients and (b) answered, “Yes”, to the question, “Did a physician or another healthcare professional ever tell you that you had high blood pressure?”.

Respondents were alleged to have received treatment if they (a) were aware of hypertension and (b) reported taking antihypertensive agents within the past two weeks.

Finally, the study participants were assumed to control their hypertension if they (a) took antihypertensive agents; and (b) their mean systolic pressure was less than 140 mmHg, while mean diastolic pressure was under 90 mmHg.

**Fig. 1:**
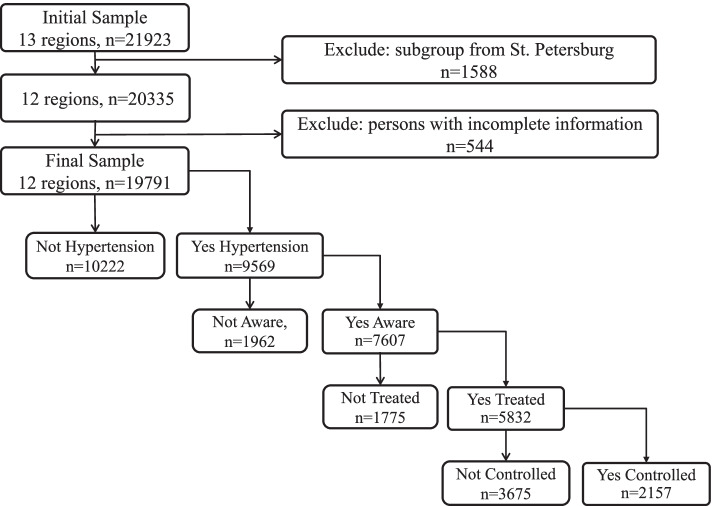
Sampling by prevalence, awareness, treatment and control of hypertension

Analytical sampling is illustrated in Fig. 1.

### Other individual-based variables

Individual-based variables included socioeconomic and demographic characteristics, such as gender, age, educational level (higher education or lower educational level), marital status (single/committed), place of residence (urban or rural area), smoking status (smoker/non-smoker), and income level. The latter was assessed indirectly via responses to three questions on the share of income spent on food, financial capabilities of the family, and perceived prosperity compared with other families. Each multiple-choice question had five answer options ranked 1 to 5 points (from the ‘poorest’ to the ‘richest’ answer option). On the basis of the cumulative score, the income level was classified into three categories: Low (3–7 points), Medium (8–10 points) and High (11–15 points). All individual-based variables were evaluated from personal interviews.

Obesity was diagnosed by the body mass index values of 30.0 kg/m2 and above.

### Characteristics of regional indices

Methods of computing and detailed description of regional indices were presented in the earlier publication [27]. In short, regional indices were calculated on the basis of 64 parameters taken from the official website of the Federal State Statistics Service of the RF characterizing various aspects of 12 RF regions under study. Most numbers on that site related to the period of 2010–2014, with the exception of gross regional product and per capita household consumption (the latter two related to 2010–2013). Principal component analysis was used as a dimensionality reduction method. As a result, five integral indices were defined (Table 1), explaining 77.6% of the total variance. Increase in Socio-Geographical Index is characterized by enlarged alcohol sales in the northern RF regions accompanied by the greater crime rate and deterioration of social living conditions (housing quality, learning environment for children). High demographic index means living in depressed regions with negative population growth rate and high proportion of elderly people in the general structure of the population. High values of industrial index incorporate living in regions with developed mining and electricity production, unfavorable working conditions for most employees, and high levels of industrial emissions into the air from stationary sources. High mixed index characterizes the geographical position of the region (degree of East longitude), along with development of fishing and fish farming, high number of paid services in the region, big number of private vehicles, and augmented proportion of women in the general structure of the population. Finally, an enlarged economic index indicates an increase in retail trade, per capita income and household consumption in the region, as well as increased manufacturing (factories, plants) and higher inequality in terms of income distribution (Gini index).

**Table 1:** Factor loadings of the principal regional indices identified

Characteristic	Factors (Indices)				
	**1**	**2**	**3**	**4**	**5**
% from total variance	28.0	16.9	12.5	10.4	9.8
Sales of vodka	0.95				
Average annual temperature	-0.88				
Timberland area	0.82				
Sales of wine-making products	0.80				
Number of recorded crimes	0.76				
Location of the regional center, north latitude	0.71				
Decrepit and dilapidated housing	0.69				
Portion of students second and third shifts	0.69				
Sales of low-alcohol beverages	0.67				
Sales of brandy and brandy spirits	0.66				
Natural increase rate		-0.99			
Crude birth rate		-0.94			
Population of unemployable age		0.92			
Crude mortality rate		0.91			
Mortality rate from diseases of the respiratory system		0.70			
Mineral extraction			0.90		
Mortality rate from tuberculosis			0.80		
Electric power production			0.79		
Mortality rate from infections			0.79		
Portion of people employed at toxic and (or) hazardous jobs			0.78		
Mortality rate from external causes			0.73		
Population size			0.71		
Emissions of pollutants into the atmosphere			0.67		
Number of employees of fisheries				0.95	
Per capita amount of paid services				0.95	
Number of private passenger cars				0.78	
Male/female ratio				-0.77	
Location of the regional center, east longitude				0.69	
Per capita retail turnover					0.92
Per capita actual final consumption of households					0.91
Gini Index					0.88
Per capita income per month					0.84
Manufacturing					0.76

### Statistical methods

Analyzed data are presented as a two-stage sample with individual-based and regional characteristics, which requires proper statistical methodology. In this regard, generalized estimating equations [28, 29] with robust standard errors were used to investigate associations between regional indices and prevalence, awareness, treatment and control of hypertension, taking into consideration nested structure of the data (study subjects in regions). Several sets of logistic models were developed with the calculation of odds ratio and Wald statistic. The null model included solely individual-based variables. Model 1 encompassed all regional indices, along with individual-based variables. Since significant interactions of gender and age of respondents with regional indices were revealed, the analysis of Model 1 was also conducted separately for these parameters. Descriptive statistics and generalized estimating equations were obtained using SPSS, version 22 (IBM Corp., USA).

## Results

General characteristics of the sample are presented in Table 2.

**Table 2:** Individual-level summary statistics, *n* = 19,791

Characteristics		Number	Percent
Hypertension outcomes	Prevalence (*n* = 19,791)	9569	48.4
	Awareness (*n* = 9569)	7607	79.5
	Treatment (*n* = 7607)	5832	76.7
	Control (*n* = 5832)	2157	37.0
Male		7599	38.4
Rural location		4069	20.6
High education		8352	42.2
Family Yes		12,783	64.6
Obesity		6429	32.5
Smoking		4306	21.8
Age	25–34 years	4165	21.0
	35 44 years	3940	19.9
	45–54 years	5528	28.0
	55–64 years	6158	31.1
Income	Low	3348	16.9
	Median	13,074	66.1
	High	3369	17.0
Region	Krasnoyarsk	1478	7.5
	Vladivostok	2058	10.4
	Volgograd	1414	7.1
	Vologda	1583	8.0
	Voronezh	1576	8.0
	Ivanovo	1780	9.0
	Kemerovo	1550	7.8
	Samara	1561	7.9
	Orenburg	1544	7.8
	Tomsk	1548	7.8
	Tyumen	1609	8.1
	Vladikavkaz	2090	10.6

In general sample, regional indices exhibited no significant associations with the prevalence of hypertension (Table 3). At the same time, the Socio-Geographical Index was directly associated with the prevalence of hypertension in men (1.18; 1.05–1.32) and in subjects 50 years of age and older (1.16; 1.02–1.32).

**Table 3:** Multivariate association of individual and regional variables with hypertension

Predictor	All sample		Stratification by Sex				Stratification by Age			
			**Women**		**Men**		** < 51 years**		** ≥ 51 years**	
	**OR**	**95% CI**	**OR**	**95% CI**	**OR**	**95% CI**	**OR**	**95% CI**	**OR**	**95% CI**
Socio-geographical index	1.11	0.99–1.25	1.07	0.95–1.21	1.18	1.05–1.32	1.06	0.94–1.20	1.16	1.02–1.32
Demographic index	1.08	0.93–1.25	1.07	0.95–1.21	1.09	0.91–1.30	1.04	0.89–1.22	1.12	0.98–1.28
Industrial index	0.94	0.83–1.07	0.90	0.79–1.02	1.01	0.88–1.15	0.94	0.82–1.08	0.93	0.82–1.06
Mixed index	0.98	0.93–1.03	0.98	0.93–1.03	0.99	0.94–1.06	0.99	0.94–1.05	0.97	0.92–1.02
Economic index	1.00	0.80–1.25	1.04	0.85–1.28	0.94	0.73–1.21	1.00	0.80–1.24	0.99	0.79–1.24

Note: adjustment for all individual variables: sex, age, location, income, education, family, obesity, smoking

The data in Table 4 show that the Demographic Index (1.13; 1.02–1.25) and the Industrial Index (1.15; 1.01–1.33) were directly associated with hypertension awareness in the general sample, but the Mixed Index was negatively associated with it (0.93; 0.88–0.99). Similar associations were detected for stratified samples, but with some subgroup differences. For example, the discovered associations were more typical for women, whereas in men, such relationships only marginally approached statistical significance, even though they were of the same direction. The associations of the Demographic Index were more pronounced in people 50 years of age and older. Both Industrial Index and Mixed Index were equally associated with hypertension awareness, regardless of age.

**Table 4:** Multivariate association of individual and regional variables with awareness

Predictor	All sample		Stratification by Sex				Stratification by Age			
			**Women**		**Men**		** < 51 years**		** ≥ 51 years**	
	**OR**	**95% CI**	**OR**	**95% CI**	**OR**	**95% CI**	**OR**	**95% CI**	**OR**	**95% CI**
Socio-geographical index	0.94	0.80–1.09	0.86	0.73–1.00	1.01	0.87–1.18	0.97	0.78–1.21	0.88	0.76–1.02
Demographic index	1.13	1.02–1.25	1.11	1.01–1.22	1.12	0.99–1.28	1.04	0.87–1.26	1.19	1.10–1.29
Industrial index	1.15	1.01–1.33	1.21	1.04–1.40	1.11	0.96–1.28	1.16	0.97–1.39	1.14	0.99–1.33
Mixed index	0.93	0.88–0.99	0.88	0.82–0.95	0.95	0.89–1.01	0.91	0.83–0.99	0.95	0.90–0.99
Economic index	0.99	0.77–1.27	0.96	0.78–1.18	1.05	0.77–1.44	0.95	0.67–1.37	1.01	0.83–1.23

Note: adjustment for all individual variables: sex, age, location, income, education, family, obesity, smoking

In general sample, hypertension treatment was inversely associated with the Socio-Geographical Index (0.76; 0.64–0.90) and the Mixed Index (0.91; 0.86–0.97), as clearly seen in Table 5. These associations were observed in all stratified samples (subgroups), although they were more pronounced in case of women, and people under 51 years of age. Besides, women demonstrated an inverse association of hypertension treatment with the Demographic Index (0.84; 0.75–0.95).

**Table 5:** Multivariate association of individual and regional variables with treatment

Predictor	All sample		Stratification by Sex				Stratification by Age			
			**Women**		**Men**		** < 51 years**		** ≥ 51 years**	
	**OR**	**95% CI**	**OR**	**95% CI**	**OR**	**95% CI**	**OR**	**95% CI**	**OR**	**95% CI**
Socio-geographical index	0.76	0.64–0.90	0.71	0.60–0.82	0.83	0.68–1.00	0.71	0.59–0.85	0.79	0.67–0.94
Demographic index	0.91	0.77–1.08	0.84	0.75–0.95	1.01	0.79–1.29	0.97	0.81–1.16	0.89	0.75–1.05
Industrial index	0.99	0.88–1.12	0.98	0.89–1.09	1.00	0.85–1.17	0.97	0.86–1.08	1.01	0.86–1.18
Mixed index	0.91	0.86–0.97	0.89	0.85–0.93	0.92	0.85–1.00	0.89	0.84–0.93	0.93	0.86–1.00
Economic index	1.11	0.86–1.44	1.04	0.85–1.27	1.23	0.89–1.69	1.14	0.84–1.54	1.10	0.86–1.40

Note: adjustment for all individual variables: sex, age, location, income, education, family, obesity, smoking

In general sample, an inverse association of the Socio-Geographical Index with the control of hypertension (0.79; 0.69–0.90) was revealed; similar associations were observed in all stratified samples (subgroups) (Table 6). No significant associations were found in the general sample for other regional indices; however, noteworthy patterns were observed in stratified samples (subgroups).

**Table 6:** Multivariate association of individual and regional variables with control

Predictor	All sample		Stratification by Sex				Stratification by Age			
			**Women**		**Men**		** < 51 years**		** ≥ 51 years**	
	**OR**	**95% CI**	**OR**	**95% CI**	**OR**	**95% CI**	**OR**	**95% CI**	**OR**	**95% CI**
Socio-geographical index	0.79	0.69–0.90	0.83	0.72–0.95	0.73	0.63–0.84	0.73	0.64–0.84	0.83	0.72–0.94
Demographic index	0.90	0.78–1.04	0.86	0.77–0.97	1.03	0.85–1.25	0.83	0.71–0.97	0.94	0.82–1.07
Industrial index	1.11	1.00–1.23	1.13	1.02–1.26	1.06	0.94–1.19	1.03	0.91–0.17	1.14	1.03–1.27
Mixed index	1.00	0.94–1.06	0.99	0.94–1.04	1.00	0.94–1.06	0.95	0.90–0.99	1.00	0.95–1.07
Economic index	0.88	0.73–1.07	0.84	0.70–1.01	0.97	0.80–1.17	0.96	0.77–1.20	0.85	0.71–1.02

Note: adjustment for all individual variables: sex, age, location, income, education, family, obesity, smoking

Demographic Index was inversely associated with the control of hypertension in women and in people under 51 years old. Industrial Index was directly associated with the control of hypertension in women and subjects 50 years of age and older. In addition, people under 51 years old exhibited the direct association of the Mixed Index with the hypertension control (0.95; 0.90–0.99).

The values of the effect criteria in Wald Chi-Squared models (Table 7) implied that the individual-based population characteristics, first of all, gender, age and concomitant diseases (e.g., obesity), had the strongest associations with the prevalence, awareness, treatment, and control of hypertension. Simultaneously, regional living conditions were less associated with the hypertension prevalence; however, the importance of this parameter was similar to significance of individual-based variables for the awareness, treatment and control of hypertension.

**Table 7:** The meaning of the model effect criteria (Likelihood Type III test, Chi-Square Wald)

Predictor	Prevalence	Awareness	Treatment	Control
Sex	19.0	32.8	47.0	21.8
Location	1.7	0.3	0.1	2.5
Income	3.8	6.0	2.2	2.6
Education	21.3	1.1	1.7	20.3
Family	10.2	0.6	0.3	1.2
Obesity	227.4	58.6	5.3	78.7
Smoking	0.6	0.1	8.1	0.1
Age	1212.5	64.2	87.2	71.4
Socio-geographical index	7.9	3.7	19.3	19.2
Demographic index	2.6	19.5	7.4	5.9
Industrial index	2.5	6.4	0.5	6.5
Mixed index	4.7	10.6	24.8	13.9
Economic index	0.4	0.2	1.6	3.3

Note: For individual variables (sex, location, income, education, family, obesity, smoking, age), the Chi-Square Wald values are taken from Model 0. For all indices, the maximum possible Chi-Square Wald values from among the models of the general sample (Model 1) are taken and stratification groups by sex, age and education

## Discussion

The results of our study showed that after adjusting for individual-based characteristics, there were significant associations of regional characteristics with the prevalence, awareness, treatment and control of hypertension. The Socio-Geographical Index demonstrated the most stable associations, and was also the only indicator exhibiting stable associations with the prevalence of hypertension. Demographic, Industrial, and Mixed Indices showed steady associations with awareness, treatment and control of hypertension, predominantly in women and/or in certain age groups.

Due to significant differences in the analyzed regional characteristics, it is difficult to compare our data with the results of other studies. Moreover, different scales of the analyzed regional characteristics should be taken into consideration. As mentioned above, most studies dealt with small territorial units – neighborhoods, districts within a single postal code, etc. Nevertheless, when discussing our results, we will attempt to compare them with the available data from other studies, however, emphasizing a somewhat conditional nature of such comparisons.

### Prevalence of hypertension

Our data implied that the Socio-Geographical Index was directly associated with the prevalence of hypertension among men and the elderly. We could try to explain the observed trend in terms of the functional description of this index: it presumes that deterioration of social living conditions, along with societal marginalization, may result in the formation of a high allostatic load [12, 30] leading, in turn, to the increased probability of high blood pressure and, consequently, to the development of hypertension. Simultaneously, our results demonstrated no associations with the prevalence of hypertension in general population, which indicated the selectivity of this effect. The results of other studies did not always confirm the negative impact of unfavorable social conditions on blood pressure and the prevalence of hypertension in the general population [21, 23, 31].

According to our results, social living conditions were associated with the prevalence of hypertension in men but not in women. This finding contradicted the results of other studies [20], including those based exclusively on the analysis of female population [32, 33]. On the other hand, an American longitudinal study demonstrated a controversial positive effect of the number of reported crimes in a residential area on the reduction of both systolic and diastolic blood pressure in women, but not in men [16]. The association of an increase in the hypertension prevalence with a deterioration of social living conditions in older people, which we have identified, was supported by other studies. In particular, the possibility was discussed that the influence of unfavorable characteristics inherent in the place of residence could be more pronounced for the elderly population, but not for the general sample [22, 34].

In our study, the Economic Index exhibited no significant association with the prevalence of hypertension, albeit some studies demonstrated a direct effect of poverty rate on blood pressure and the prevalence of hypertension [35–37]. The only published large-scale study we have found analyzed the impact of income inequality in terms of Gini index at the departmental level on hypertension in Colombia [17]. In contrast, in our study, Gini index was included in the Economic Index. In adjusted models, women (but not men), living in the areas with high income inequality, were most likely to have hypertension. A number of studies had similar results regarding the absence of associations between hypertension and economic characteristics of living conditions [13, 38].

### Awareness, treatment and control of hypertension

Our results implied that an unfavorable social environment (Socio-Geographical Index) was associated with a decrease in the self-reported medication use and proper control of arterial hypertension in the general sample. Both Demographic and Industrial Indices were directly and most strongly associated with hypertension awareness. To a greater extent, this was observed in women, as well as in certain age groups. Besides, different gender and age groups demonstrated augmented control of hypertension with a decrease in Demographic Index and, conversely, with increase of the Industrial Index. This direct relationship among the regional industrial development, and awareness and control of hypertension, could be explained by the higher attention of large public and private industrial corporations paid to the health of their employees than was the general trend applying to the entire population. There are very few studies on the impact of regional characteristics in terms of living conditions on the awareness, management and control of hypertension. Overall, these studies have shown trends of reduced awareness, treatment and control of hypertension in poor living conditions [13, 21, 22, 39, 40]. In contrast, our results suggest somewhat more complex relationships between awareness, treatment, hypertension control, and regional living conditions.

### Advantages and limitations of the study

With the exception of the aforementioned Colombian project [17], our research is the only study analyzing the associations of the specific features of the population, living within large territorial units, with the prevalence of hypertension. Moreover, we have found no available literary sources with similar analysis of awareness, treatment and control of hypertension. This is despite the fact that there are studies concerning other diseases, mortality or behavioral risk factors in large areas (states or provinces). This is despite the fact that studies of other diseases, related mortality, or behavioral risk factors were conducted over large areas (states or provinces).

We evaluated regional characteristics employing the empirical approach rarely used in similar studies. In particular, we found just one similar study on the prevalence of hypertension [13].

Our results are based on a large sample with data processing via contemporary statistical approaches, allowing adequate analysis of hierarchical samples.

Finally, the results of our study represent the pioneering attempt in the history of Russian research to understand why there are such substantial differences in the prevalence, awareness, treatment and control of hypertension between the RF regions.

Regarding the shortcomings of our study, we would like to point out the difficulties of interpreting some obtained results. This was the case for the Mixed Index: the latter demonstrated fairly stable associations that were difficult to interpret. We hope that further research would help resolving this issue. Another limitation arose from one of the stated advantages: a very small number of studies similar to ours caused the complexity in interpreting the established associations in general. For example, no one ever considered the social wellbeing of the territory of residence in terms of sales or consumption of alcoholic beverages by its population.

## Conclusion

Our study evaluated the associations of the vital characteristics inherent in population of large RF regions with the prevalence of arterial hypertension, as well as with awareness, treatment and control of this disease. It was found that the association of regional living conditions with the prevalence of hypertension is relatively weak, compared to predictors at the individual level, but this influence is important for awareness, treatment and control of hypertension. These patterns describe an empirical approach (i.e., a posteriori approach) to the analyzed regional characteristics. There are very few available published sources on the geography of hypertension using the case studies of large areas and/or using a posteriori approaches similar to ours. Therefore, our results provided novel, original insights from the standpoint of cardiovascular disease epidemiology in the RF, as well as in the context of investigating the impact of living conditions on population health.

## Data Availability

The datasets, analyzed in the course of our study, are not publicly available due to protection of the privacy of subjects participating in the study. However, they are available from the corresponding author on reasonable request.

## Abbreviations

CVD: Cardiovascular diseases
ESSE-RF: Epidemiology of Cardiovascular Diseases and Risk Factors in the Regions of the Russian Federation study
OR: Odds ratio
CI: Confidence interval
